# Reverse Genetics Approaches for the Development of Influenza Vaccines

**DOI:** 10.3390/ijms18010020

**Published:** 2016-12-22

**Authors:** Aitor Nogales, Luis Martínez-Sobrido

**Affiliations:** Department of Microbiology and Immunology, University of Rochester, Rochester, NY 14642, USA

**Keywords:** influenza vaccines, influenza virus, reverse genetics, live-attenuated influenza virus, influenza inactivated virus, recombinant influenza virus, vaccination, antivirals, universal vaccines

## Abstract

Influenza viruses cause annual seasonal epidemics and occasional pandemics of human respiratory disease. Influenza virus infections represent a serious public health and economic problem, which are most effectively prevented through vaccination. However, influenza viruses undergo continual antigenic variation, which requires either the annual reformulation of seasonal influenza vaccines or the rapid generation of vaccines against potential pandemic virus strains. The segmented nature of influenza virus allows for the reassortment between two or more viruses within a co-infected cell, and this characteristic has also been harnessed in the laboratory to generate reassortant viruses for their use as either inactivated or live-attenuated influenza vaccines. With the implementation of plasmid-based reverse genetics techniques, it is now possible to engineer recombinant influenza viruses entirely from full-length complementary DNA copies of the viral genome by transfection of susceptible cells. These reverse genetics systems have provided investigators with novel and powerful approaches to answer important questions about the biology of influenza viruses, including the function of viral proteins, their interaction with cellular host factors and the mechanisms of influenza virus transmission and pathogenesis. In addition, reverse genetics techniques have allowed the generation of recombinant influenza viruses, providing a powerful technology to develop both inactivated and live-attenuated influenza vaccines. In this review, we will summarize the current knowledge of state-of-the-art, plasmid-based, influenza reverse genetics approaches and their implementation to provide rapid, convenient, safe and more effective influenza inactivated or live-attenuated vaccines.

## 1. Influenza Virus

### 1.1. Influenza Virus Structure and Genome Organization

Influenza A (IAV) and B (IBV) viruses belong to the *Orthomyxoviridae* family of enveloped viruses [1]. IAV is able to infect several species and mostly exists in the wild aquatic fowl reservoir [2,3,4]. On the other hand, IBV is mainly restricted and adapted to humans, although sporadic infections of seals have been documented [5,6].

**Figure 1:**
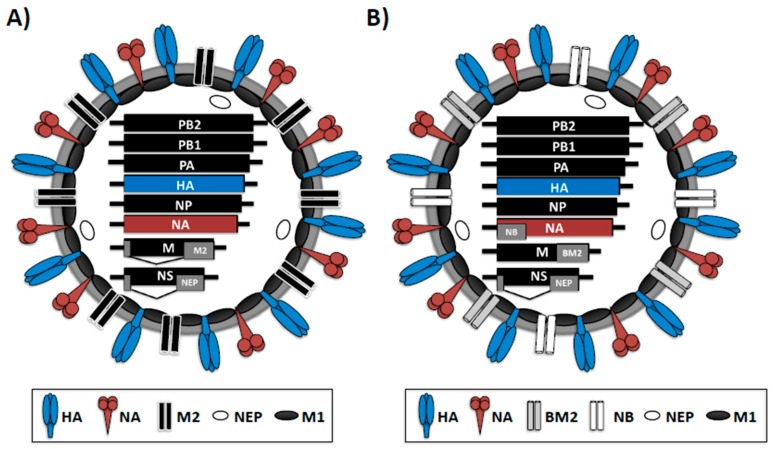
Virion structure of IAV (**A**) and IBV (**B**): IAV and IBV are surrounded by a lipid bilayer containing the two viral glycoproteins hemagglutinin (HA), responsible for binding to sialic acid-containing receptors in the surface of susceptible cells, and neuraminidase (NA), responsible for viral release from infected cells. Furthermore, in the virion membrane is the ion channel M2 (IAV) or BM2 and NB (IBV) proteins. Under the viral lipid bilayer is a protein layer composed of the M1 protein, which plays a role in virion assembly and budding, and the nuclear export protein (NEP) involved in the nuclear export of the viral ribonucleoprotein (vRNP) complexes. The eight viral segments and protein products are indicated inside the virions. Black lines at the end of each of the eight IAV and IBV vRNAs indicate the 3′ and 5′ non-coding regions (NCR). PB1 and PB2, polymerase basic 1 and 2; PA, polymerase acid; NP, nucleoprotein; NS, nonstructural gene; M: matrix; BM2: influenza B matrix protein 2.

IAV and IBV genomes contain eight negative sense, single-stranded viral (v)RNA segments [1] (Figure 1). IAV and IBV vRNAs contain a central coding region that is flanked at both terminal ends by non-coding regions (NCRs), which serve as promoters to initiate genome replication and gene transcription by the viral polymerase complex [1,7]. Influenza vRNAs in the virion are found as viral ribonucleoprotein (vRNP) complexes encapsidated by the viral nucleoprotein (NP) and a single copy of the viral polymerase complex. Influenza virus-encoded RNA-dependent RNA polymerase (RdRp) [8] is a trimeric complex consisting of the polymerase basic 1 (PB1) and 2 (PB2) and acidic (PA) proteins and, together with the viral NP, are the minimal components involved in viral replication and transcription [9].

IAV and IBV share many features, but they differ in their host range, virion structure, genomic organization and glycan binding specificities [1,10]. Despite having similar genomes encoding homologous proteins, IAV and IBV are distinguished by the different lengths of proteins and non-coding regions (NCRs) that serve as promoters for genome replication and gene transcription [5,11,12] (Figure 1). Likewise, they can also be distinguished by the presence of accessory proteins encoded from overlapping open reading frames (ORFs) and by the antigenic differences of internal proteins [13] (Figure 1A,B). For instance, IAV and IBV both encode ion channel proteins from the gene M segment 7, M2 and BM2, respectively. The M2 and BM2 proteins of IAV or IBV are encoded together with the matrix protein 1 (M1) and both are incorporated into virions and expressed on the surface of virus-infected cells [1]. However, the M2 protein of IAV is translated from a spliced mRNA [14], while the IBV BM2 protein is translated using a different strategy, where the initiation codon of BM2 protein overlaps the termination codon of M1 protein (UAAUG, a stop-start pentanucleotide) [15]. In addition, IBV expresses the NB ion channel, which is absent in type A influenza virus [1] (Figure 1B). However, both influenza viruses encode two surface glycoproteins, hemagglutinin (HA) and neuraminidase (NA) (Figure 1A,B). IAV and IBV HA proteins are involved in binding to cellular receptors and responsible for the fusion of the viral and endosomal membranes [16]. Infection with IAV or IBV induces a protective immunity mediated, at least partially, by antibodies directed against the viral HA, which is the main immunogenic target in both natural infections and vaccine approaches. Influenza NA glycoprotein is responsible for the cleavage of sialic acid moieties from sialyloligosaccharides and facilitates the release of newly produced virions from infected cells [17,18]. IAVs are classified on the basis of the antigenic properties of HA and NA into 18 HA (H1–H18) and 11 NA (N1–N11) subtypes [1,19,20]. However, only IAV H1N1 and H3N2 subtypes are currently circulating in humans. On the other hand, two major lineages of IBV are circulating in humans, the Victoria-like and Yamagata-like subtypes that are divergent from the ancestral IBV (B/Lee/1940) and have been co-circulating in humans since the 1980s [5,21,22]. These two subtypes are the predominant circulating virus strains about once every three years [23,24,25].

### 1.2. Influenza Life Cycle

Infection with influenza viruses begins when the viral HA protein binds to its cellular receptor, a sialylated glycoprotein containing α-2,3 or α-2,6 linkages [16]. Upon the binding to the receptor, the uptake of the virus by receptor-mediated endocytosis is initiated, and the cell membrane engulfs the virus particles in an endosome. After endocytosis and upon acidification of the endosome, influenza viral HA undergoes a conformational change responsible for the fusion of the viral and the endosome membrane [16]. Then, the M2 (IAV) or BM2 (IBV) ion channel proteins promote the release of the vRNP complexes from the virion core into the cytoplasm of infected cells [14,26]. The vRNPs are translocated from the cytoplasm to the nucleus of infected cells to initiate viral genome replication and gene transcription [27]. The nuclear export (NEP) and matrix 1 (M1) proteins are responsible for the nuclear export of newly-synthesized vRNPs into the cytoplasm of infected cells. Notably, a single copy of each of the eight vRNAs is packaged into new virions [28,29]. Selective package of influenza vRNAs into nascent virions is mediated by RNA-RNA interactions of vRNA packaging signals present at the terminal ends of each of the vRNA segments [30]. Finally, the receptor-destroying enzymatic activity of NA is responsible for the release of newly-synthesized viral particles from the surface of infected cells [17].

Among the first battles between the host and the virus, cellular type I interferon (IFN-I) plays an important role in controlling viral infection [31]. Therefore, viruses have developed multiple strategies to hijack the host cellular immune response. Influenza vRNA segment 8, or the nonstructural (NS) gene, encodes two distinct proteins through a direct or alternative splicing mechanism [1]. Influenza virus segment 8 produces the nonstructural protein 1 (NS1) as a primary transcript, whereas NEP is produced by alternative splicing of the NS mRNA [32]. NS1 has multiple functions during the replication cycle of influenza virus, but most notably inhibits induction of IFN-I response and innate immune activation [31].

### 1.3. Influenza Viruses and Their Impact on Human Health

Influenza viruses pose a threat to human health and are responsible for global epidemics every year [33,34,35,36,37]. In fact, influenza virus is one of the most significant causes of morbidity and mortality yearly, leading to a significant economic impact [38]. Despite the implementation of effective and comprehensive vaccination programs, the World Health Organization (WHO) estimates that seasonal influenza virus infections results in about one billion infections, 3–5 million cases of severe disease and between 300,000 and 500,000 deaths around the world annually [39]. Moreover, just in the United States (U.S.), influenza viral infections result in an average of 87 billion dollars of cost due to prophylactic, therapeutic and hospitalization costs, as well as missed school or work days [38,40,41,42]. In addition to seasonal influenza, IAV can cause sporadic pandemics of great consequences when novel viruses are introduced into humans [43]. The mechanisms responsible for the emergence of seasonal and pandemic IAVs are antigenic drift and antigenic shift, respectively [12,44,45,46]. In the case of seasonal influenza, mutations in the viral genome result in the selection of antigenic variants with changes in viral tropism, increased levels of viral fitness or in the ability to escape neutralizing antibody (NAb) responses induced upon previous natural infections or vaccinations [44,45]. Moreover, antigenic drift can lead to variant viruses resistant to antivirals [47]. On the contrary, antigenic shift results from co-infection of a host cell or organism with two or more IAVs where vRNAs in viral progenies are reassorted [12,46]. By obtaining viral segment constellations that confer influenza virus optimal replication, transmissibility and immunologic escape, reassortant IAVs can lead to a pandemic in immunologically-naive populations. IAVs and IBVs can reassort intratypically between subtypes or lineages, but intertypic reassortment, or genetic swapping of segments between IAV and IBV, has not been reported [21,48,49]. The absence of intertypic reassortment is mediated by the lack of compatible packaging signals between IAV and IBV [12]. In the last century, three IAV pandemics have occurred: the H1N1 Spanish flu of 1918, the H2N2 Asian flu of 1957 and the H3N2 Hong Kong flu of 1968 [44,50]. Of these three, the 1918 H1N1 Spanish flu was particularly fatal and responsible for approximately 50 million deaths around the world [51]. Although not classified as true pandemics, three epidemics of influenza H1N1 viruses in 1947, 1976 and 1977 were feared to have pandemic potential. In 2009, a swine-origin H1N1 IAV was responsible for the first influenza pandemic of the 21st century and infected, in less than one year, more than 600,000 individuals around the world [52,53]. Although influenza virus infections are primarily spread by person-to-person transmission via aerosolized droplets, infection with new avian- or swine-origin IAV can take place and may have great risk for pandemic potential if the virus acquires the ability to be transmitted between humans [54,55]. IBVs usually contribute less to seasonal epidemics than IAVs of the H3N2 subtype, but they contribute more than type A H1N1 influenza strains and are the predominant circulating virus strains once every three years [23,24,25]. Moreover, during the last decade, IBV has been the cause of several acute respiratory illness outbreaks [56,57,58,59,60]. To date, no influenza pandemics have been linked to IBVs.

### 1.4. Current Strategies to Combat Human Influenza Infections

Public health concerns posed by influenza virus infections are aggravated by the ability of influenza viruses to efficiently transmit and the limited therapeutic options to treat viral infections [37]. Thus, vaccination remains our best medical intervention to protect humans against seasonal influenza virus. However, the efficiency of current influenza vaccines is suboptimal [61]. The segmented genome of influenza viruses provides an evolutionary advantage of reassortment, or the exchange of viral genome segments between different viral strains within the same type. In addition to this exchange of genome material (or antigenic shift), influenza viruses can introduce mutations in their genomes (or antigenic drift), leading to viral mutants with resistance against current antivirals or NAbs [62,63,64].

#### 1.4.1. Influenza Vaccines

Because of the antigenic drift, influenza vaccines need to be reformulated yearly to ensure that the HA and NA present in the vaccine match those present in circulating seasonal viruses. To date, three types of influenza virus vaccines are approved by the Food and Drug Administration (FDA) for human use: recombinant viral HA, inactivated virus and live-attenuated viruses [65,66,67,68,69]. Regardless of the type of vaccine, seasonal influenza vaccines contain antigens from the three circulating influenza virus strains: IAV subtypes H1N1 and H3N2; and IBV (Victoria-like or Yamagata-like lineage) [67,70]. Recently, to improve the efficacy of seasonal influenza vaccines, a quadrivalent influenza vaccine formulation that includes both IBV lineages components (Victoria-like and Yamagata-like lineage) has been approved by the FDA [14].

The most common influenza vaccine is the inactivated influenza vaccine (IIV). IIV, which is administered intramuscularly, has been shown to elicit protective humoral immunity by producing NAbs that target epitopes on HA [61,71]. In contrast to IIV, the live-attenuated influenza vaccine (LAIV) and its administration mimic the natural route of virus infection, which consequently having risks and benefits [72]. An advantage is that LAIV elicits both more rapid and efficient innate and adaptive immune responses [65] and can provide more efficient cross-reactive T-cell-mediated protection against heterologous influenza viruses [72,73]. Although both IIV and LAIV have been shown to be efficient for the treatment of influenza viral infections, there is an unmet need to increase the effectiveness of seasonal influenza vaccines. Likewise, there is an urgent need to develop effective vaccines for the treatment of potential pandemic influenza viruses.

#### 1.4.2. Influenza Antivirals

Although vaccination is the main method to prevent influenza infections in humans, antivirals offer an additional countermeasure against new rapidly-spreading and/or potentially pandemic influenza viruses [74,75,76]. Therapeutic choices to control influenza infection are currently limited to two classes of FDA-approved antivirals targeting either the viral M2 ion channel (amantadine, rimantadine) [77,78,79] or the sialidase active site of NA (oseltamivir, zanamivir) [80]. The first inhibits the initial steps of the virus life cycle, while the second inhibits virus release. However, NA inhibitors are the only type of antivirals approved for the prophylaxis and treatment of IBV infections [81], although data from clinical trials have shown that oseltamivir is less effective against IBVs than against IAVs [82]. Importantly, mutations in the viral genome can lead to influenza antivirals being ineffective, like in the case of M2 blockers, which are no longer recommended against circulating seasonal influenza viral strains [83]. Therefore, the emergence of drug-resistant influenza variants is an increasing concern for controlling influenza infections [76,84], and there is a significant need for the identification of novel compounds with antivirals properties.

## 2. Influenza Vaccine Production

### 2.1. Vaccine Strain Selection

Influenza viruses evade human pre-existing immunity by accumulating mutations (antigenic drift), allowing reinfection of individuals previously exposed to natural infections or vaccinated. Thus, vaccine companies have to reformulate the composition of influenza vaccines yearly to ensure a good match between the viruses present in the vaccine and those seasonally circulating in humans [33,34,85]. The WHO Global Influenza Surveillance Network (GISN) [35], which includes more than 120 National Influenza Centers in over 90 countries [86], tracks the evolution and epidemiology of influenza viruses and uses the collected data for the vaccine strain selection process. These studies also help to understand virus evolution and epidemiology in different geographic areas [19]. Each center collects and analyzes samples of seasonal influenza viruses for communication to the WHO Collaborating Centers for Reference and Research on Influenza: (1) The Centers for Disease Control and Prevention (CDC) in Atlanta, USA; (2) The National Institute for Medical Research in Mill Hill, U.K.; (3) The WHO Collaborating Centre for Reference and Research on Influenza in Melbourne, Australia; and (4) The National Institute of Infectious Disease in Tokyo, Japan. Basically, the degree of immunity induced by one influenza strain that is effective against another strain is mainly dependent on the antigenic difference between both viral strains [87,88]. The influenza viral glycoprotein HA, the primary target of the protective neutralizing immune responses [88,89], is the focus of influenza virus surveillance and the primary component targeted by currently licensed influenza vaccines [72,89].

Influenza vaccine production is challenging because of the wait time that is needed to identify the predominant circulating virus and the production time that is needed to manufacture the vaccine. The National Influenza Centers perform virus isolation on certain samples obtained from patients to identify circulating viruses and to determine if the viruses grow efficiently in culture. Normally, influenza viruses are isolated using Madin Darby canine kidney (MDCK) cells instead of chicken embryonated eggs because higher isolation rates, especially for H3N2 IAV strains, have been reported using MDCK cells [90,91,92]. Although most influenza viruses are isolated from mammalian cells, viruses must be generated in embryonated eggs for vaccine manufacturing due to regulations [93]. Nowadays, the vaccine strains must be selected almost 7–9 months ahead of the influenza season in which they will be used. The recommendation for the strains included in the vaccine composition for the Northern Hemisphere is made in February to allow time for the ~300 million doses of vaccine to be produced in time for vaccinating people in October/November. This allows for influenza season preparation, which typically peaks sometime between December and March [86]. On the other hand, for the Southern Hemisphere, recommendations are provided in September, and vaccination takes place in March/April of the following year [86].

### 2.2. Types of Influenza Seasonal Vaccines

Seasonal influenza vaccines must protect against H1N1, H3N2 and B viral strains currently circulating in humans globally [34,86]. The main goals of influenza vaccines are the protection against infection and disease caused by influenza infections and to restrict virus transmission within the population [67,94]. NAb responses, commonly assessed by measuring hemagglutination inhibition (HAI) titers, are currently used as a serological marker of the efficient immunological response to the vaccine. The effectiveness of influenza vaccines is variable and usually higher in children and in healthy adults under the age of 65. In individuals above 65 years of age, lower effectiveness has been observed [61,71,95]. Recommendations for influenza vaccination differ between countries, but since influenza vaccines do not induce long-lasting antibody protection, annual influenza vaccinations are recommended. Nowadays, the most used influenza vaccines can be divided into IIV and LAIV [65,66,72,73,96].

#### 2.2.1. Influenza Inactivated Vaccine (IIV)

**Figure 2:**
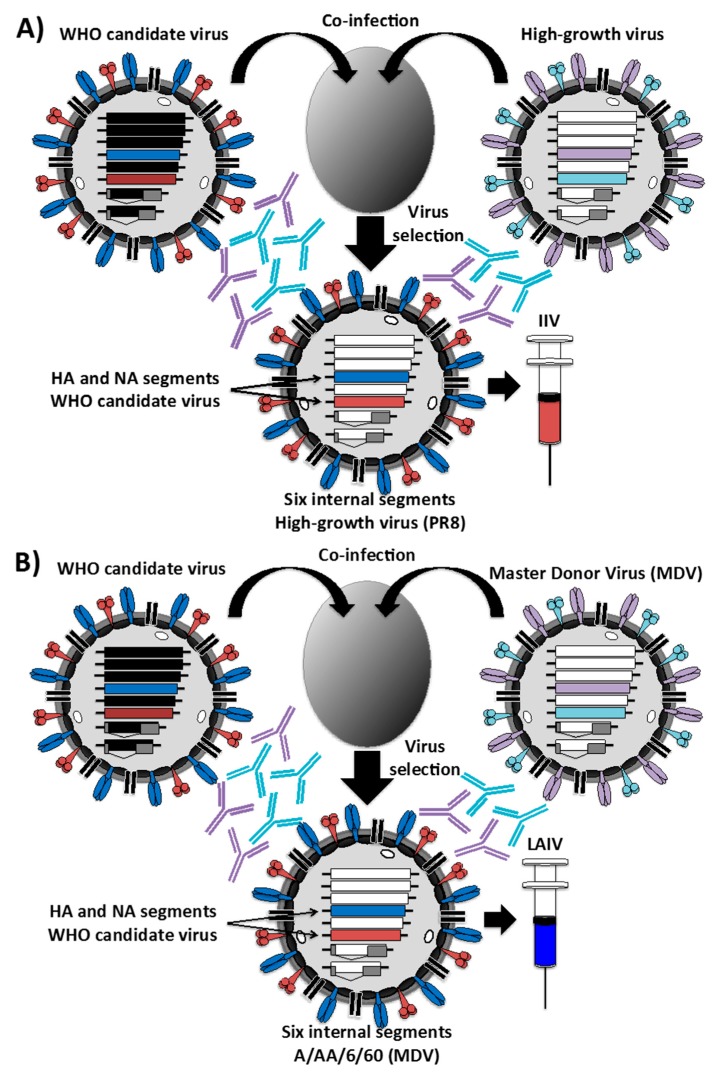
Schematic representation to produce inactivated (**A**) or live-attenuated (**B**) influenza vaccines by genetic reassortment in embryonated eggs: The traditional method for generating reassortant virus is based on the coinfection of two influenza viruses in eggs. Both the WHO candidate virus and the high-growth virus for influenza inactivated vaccine (IIV) (**A**) or the master donor virus (MDV) for live-attenuated influenza vaccine (LAIV) (**B**) are inoculated in eggs followed by the selection of appropriate seed viruses by amplification in the presence of antibodies against the HA and NA of the high-growth virus (**A**) or the MDV (**B**). The resulting viruses containing the HA and NA segments from the WHO-recommended strain and the six internal vRNAs of the high-growth virus (**A**) or the MDV (**B**) are used for vaccine production. PR8, Puerto Rico/8.

Killed virus vaccines or IIV are generally administered intramuscularly and can be classified as whole virus vaccines or split vaccines [66,71,97,98]. Whole virus vaccines were the first to be developed. The influenza virus is grown in embryonated chicken eggs, subsequently purified, concentrated and chemically inactivated with formaldehyde [98] (Figure 2). Whole virus vaccines are safe and well tolerated, with an efficacy of 60%–90% in children and adults. On the other hand, the split-virus vaccine exposes all viral proteins and subviral elements upon dissociation of the virions by a nonionic detergent treatment step [97,99]. Most influenza vaccines in the U.S. and Europe are egg-produced, formaldehyde-inactivated, then chemically disrupted with nonionic detergents after purification.

Unlike virus-based vaccines, subunit influenza vaccines consist of purified viral HA or HA/NA proteins without the other viral components [68]. These subunit vaccines can be produced in eggs if the viral proteins are prepared from viruses where HA/NA have been purified by removal of other viral component. In addition, the subunit vaccines can be generated using novel manufacturing technologies [98,100], which allows for the production of large quantities of the viral HA using baculovirus expression systems and recombinant DNA technologies [100].

The IAV strains that are used in vaccine manufacture are high-growth 6 + 2 reassortants containing the HA and NA gene segments from the target strains in the backbone of influenza A/Puerto Rico/8/1934 H1N1 (PR8) or other high growth virus. Influenza PR8 replicates at high titers in eggs and cells and also has a favorable safety profile in humans [98,101] (Figure 2A). To generate the reassortant viruses, eggs are co-infected with PR8 and seasonal strains (Figure 2A). Selection of appropriated seed vaccine viruses is made by amplification in the presence of PR8 HA and NA NAbs [98] (Figure 2A). Then, selected viruses are cloned and sequenced for confirmation. The IBV vaccines are typically wild-type (WT) viruses. However, IBV reassortants are used if WT IBV growth properties are not optimal for efficient growth and vaccine production [98].

#### 2.2.2. Live-Attenuated Influenza Vaccine

The remaining class of vaccines consists of live-attenuated influenza viruses. Attenuated human LAIVs were developed in the 1960s by serial passage of the virus in eggs using suboptimal conditions of temperature. The resulting attenuated viruses displayed a temperature-sensitive (ts) cold-adapted (ca) attenuated (att) phenotype that grew at 25 °C, but not at temperatures found in the lower respiratory tract (>35 °C) [102,103,104,105]. Because this ts, ca, att phenotype restricts virus replication to the upper respiratory track, these viruses could induce local protective immunological responses [65,95,96,98].

LAIV have been available in the U.S. since 2003 and are administrated intranasally. The advantages of a live virus vaccine, as compared to the inactivated virus vaccine, is that it is applied to the nasal mucosa where the vaccine can induce local immunity (including NAbs), generate a cell-mediated immune response and provide a cross-reactive and longer lasting immune response [106]. The current LAIVs consist of the internal viral segments (PB2, PB1, PA, NP, M and NS) of an attenuated master donor virus (MDV) and the HA and NA viral segments from the selected seasonal virus strain (Figure 2B). The MDVs used are A/Ann Arbor/6/60 (H2N2) and B/Ann Arbor/1/66 for IAV and IBV, respectively [103,104,107,108,109,110,111,112,113]. Similar to MDV IAV, MDV IBV was originally derived by serial passage of the parental WT virus and isolated at successively reduced temperatures in primary chicken kidney (PCK) cells [114]. The resulting IBV MDV grows efficiently at 25 °C (ca), but its growth is restricted at 37 °C (ts). The genetic changes in the MDV strains have been recently characterized. The MDV IAV includes five mutations in two of the viral polymerases (PB2 N265S; and PB1 K391E, D581G and A661T) and NP (D34G) [103,104,115]. The MDV IBV has been reported to contain two mutant amino acids in NP (A114 and H410) and one in PA (M431) that are responsible for the ts, ca signature [108]. Two additional residues in M1 (Q159 and V183) provide the MDV IBV an attenuated (att) phenotype [108]. Although LAIVs have been approved for clinical use, to date, their mechanism of attenuation has not been completely understood. However, the tolerability of LAIVs in specific populations is an important concern because of the inherent risk of immunizing with live viruses. Thus, LAIVs are not recommended for immunocompromised patients or asthmatics [116] and are not approved for use in children under two years of age [72,117]. Moreover, LAIVs harboring different HA and NA viral segments can be unequally safe or immunogenic from year to year as the viral HA and NA are different. Similar to U.S. LAIVs, in Russia, two ts, ca MDV IAVs have been obtained by using a similar temperature adaptation approach. The two Russian MDVs were originated from the same parental A/Leningrad/134/57 H2N2 (Len/57) influenza strain [118,119]. Similarly to the U.S. MDV, the Russian MDVs have been selected by growing Len/57 in embryonated chicken eggs at lower (25 °C) temperatures. The MDV Len/17 was obtained after 17 passages of Len/57 at 25 °C and has been used in the preparation of the Russian LAIV for use in adults. The genetic changes in the MDVs Len/17 have been identified, and these include four mutations in three viral proteins (PB2 V478L; PB1 K265N and V591I; NEP M100I) [120]. The second donor strain (Len/47) was obtained after a total of 47 passages and has been used for vaccinating children less than 16 years of age, although the genetic changes responsible for the further ts, ca phenotype have not been well characterized [121].

In all cases, like for IIV, the MDVs contain six internal genes to generate vaccine strains in combination with the seasonal recommended HA and NA genes from the circulating strains (Figure 2B). LAIVs are generated either by classical reassortment in eggs (as previously described for the IIV) (Figure 2B) or by reverse genetics, as indicated below [98].

### 2.3. Substrates for the Production of Influenza Vaccines

Most of the influenza virus vaccines have been traditionally produced in eggs, but with the progress made in mammalian cell culture technologies, influenza vaccine manufacturers have invested in these novel cell culture systems for the mass production of influenza vaccines without the need of eggs.

#### 2.3.1. Chicken Embryonated Eggs

The majority of the currently licensed influenza vaccines that are made by biotechnology companies use fertilized chicken egg-based production technology, but this process has multiple drawbacks. This form of manufacturing depends on the access to embryonated eggs, relies on the ability of influenza viruses to efficiently grow in eggs and is a resource- and time-intensive process [91,122,123,124]. Moreover, the risk of egg contamination by avian pathogens or microbes represents a risk for the production of influenza vaccines [125,126]. Importantly, in the case of an IAV pandemic, the egg supplies can be compromised. For IIV, one dose for adults contains approximately 45 µg of HA (15 µg of viral HA for each of the three antigenic H1N1, H3N2 and IBV components), meaning one egg = 7–10 dose of vaccine. LAIVs share similar egg-based production process steps. The LAIV is recovered from infected eggs and then purified and concentrated [90]. Importantly, new vaccine production approaches that do not depend on the propagation of influenza viruses in eggs (e.g., cell cultures) represent an excellent option to increase influenza vaccine production.

#### 2.3.2. Cell Cultures

Mammalian cell cultures have been used in the biopharmaceutical industry for the production of therapeutic proteins and/or vaccines [127,128]. In 2012, the FDA approved a cell-based production process for influenza vaccines, but the manufacturing process begins with egg-grown vaccine viruses per FDA regulations. Influenza vaccine production using FDA-approved MDCK or Vero (African green monkey kidney) cells may eventually and completely replace the use of eggs for the production of influenza vaccines in the future. Influenza vaccine production in mammalian cell lines offers several advantages over egg-based production: it allows faster and greater production capacity, improved availability of substrate for virus growth [122,127] and eliminates reliance on the supply of embryonated chicken eggs [129,130]. In addition, cell cultures can be cryopreserved and scaled up in bioreactors at any time.

### 2.4. Adjuvants

Adjuvants have been shown to enhance the immune response elicited by an antigen and could be used to improve the immunogenicity of IIV [131,132]. The use of adjuvants could also reduce vaccine dose, stretching antigen and vaccine supplies. Currently, FDA-licensed adjuvants for influenza vaccine usage include aluminum salt (alum) and the squalene oil-in-water emulsion systems MF59 (Wadman 2005 (Novartis)) [133] and AS03 (GlaxoSmithKline) [134]. However, most of the current IIVs do not contain any type of adjuvant, but many are under investigation.

## 3. Influenza Reverse Genetics for Vaccine Development

### 3.1. Influenza Reverse Genetics

Genetics techniques to generate recombinant viruses were first developed for DNA viruses and based on the transfection of cells with plasmids encoding the viral genome or by heterologous recombination between plasmids bearing viral sequences with the virus genome and the helper virus [135]. Initial genetics approaches for DNA viruses were followed by manipulations of positive-sense RNA viral genomes [136,137]. Transfection of plasmid DNA, or RNA transcribed directly in vitro from plasmids, containing the genome of poliovirus into susceptible cells led to the generation of recombinant infectious poliovirus [138]. However, the genomes of negative-sense RNA viruses, including influenza, were less suitable to molecular biology manipulations in comparison with DNA or positive-sense RNA viruses since their genomes are complementary to mRNA in their orientation and, therefore, not infectious by themselves [1,7]. They require the presence of vRNA(s) and the viral RdRps to initiate the replication cycle of the virus [33,44]. The advent of reverse genetics and molecular engineering has transformed the influenza field, allowing multiple questions to be answered using genetically-engineered recombinant influenza viruses [139]. Such studies include mechanisms of viral genome replication and gene transcription, pathogenicity and virulence, virus-host interactions or host range and transmissibility [12,13,139,140]. Moreover, these technologies have been implemented to develop influenza vaccines [141] and to generate recombinant influenza viruses expressing foreign proteins as vaccine vectors [142,143,144] or harboring reporter genes to easily track viral infections [142,145,146,147].

**Figure 3:**
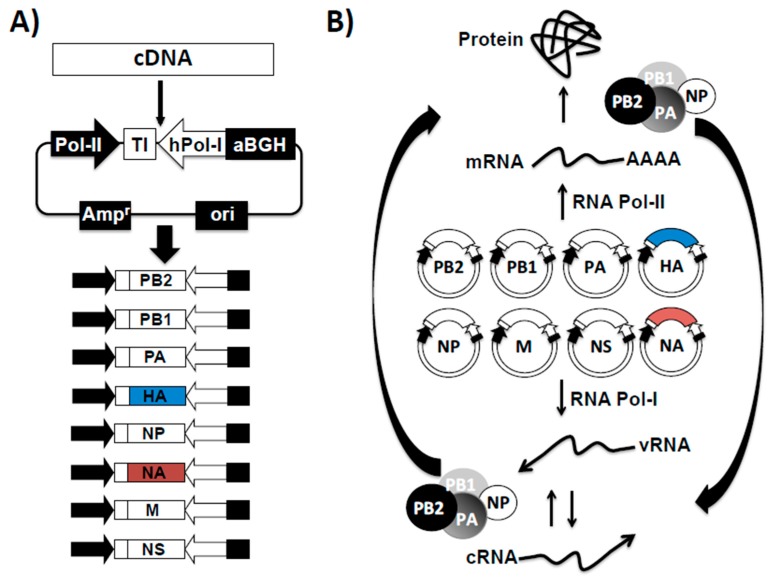
Influenza vRNA cloning into bi-directional rescue plasmids. (**A**) Schematic representation of an ambisense plasmid, influenza cDNA inserts and generation of influenza rescue plasmids: The ambisense plasmid is a bi-directional vector containing the human polymerase I promoter (hPol-I, white arrow) and the mouse Pol-I terminator (TI, white box) sequences to direct the synthesis of the influenza vRNAs. Transcription from the Pol-I cassette results in vRNAs identical to those present in influenza virus, allowing their recognition by the influenza polymerase complex. In opposite orientation to the Pol-I cassette, a polymerase II-dependent cytomegalovirus promoter (Pol-II, black arrow) and a polyadenylation sequence (aBGH, black box) direct the synthesis of influenza proteins from the same viral cDNAs; (**B**) Influenza plasmid-based reverse genetics: In cells transfected with the influenza ambisense plasmids, the Pol-I cassette generates the eight negative sense vRNAs (**bottom**) while the Pol-II directs the synthesis of the eight viral mRNAs (**top**) that are translated into the influenza viral proteins. After translation, influenza NP and polymerase complex PA, PB1 and PB2 associate with the vRNAs to form the viral ribonucleoprotein (vRNP) complexes and initiate transcription from the viral promoter located within the non-coding regions at the 3′ termini of the vRNAs. Transcription results in the synthesis of more mRNAs and proteins. The influenza polymerase complex also replicates the vRNAs into complementary (c)RNAs that serve as templates for the amplification of vRNAs. Newly-synthesized vRNAs, together with the structural viral proteins result in the formation of new influenza viruses. Blue and red boxes indicates the HA and NA of seasonal influenza viruses to be included in either the IIV or the LAIV vaccine, respectively. Amp^r^: ampicillin resistance gene; Ori: plasmid origin of replication.

Plasmid-based reverse genetics for influenza virus allows for the simultaneous expression of the viral components involved in viral genome replication and gene transcription (PB2, PB1, PA and NP) and the eight negative-stranded vRNAs in transfected susceptible cells, which together generate de novo, recombinant IAVs or IBVs (Figure 3) [48]. The goal to generate vRNA in vivo from cloned complementary (c)DNAs was achieved when the RNA polymerase I (Pol I) system for influenza vRNA synthesis was established [148,149]. Pol I is a nuclear enzyme that transcribes ribosomal (r)RNA, which like influenza vRNA does not contain a cap structure on the 5′ or poly (A) structures on the 3′ ends [150,151]. Importantly, Pol I initiates and terminates transcription at defined promoter and terminator sequences allowing the generation of vRNAs without additional nucleotides at their 5′ or 3′ ends, which is required for efficient generation of recombinant viruses using reverse genetics. Nevertheless, the Pol I promoter is species specific [152,153] and was originally established for influenza rescue in human cells [135,140,149,154]. Currently, the Pol I promoters of different species have been identified, allowing the generation of recombinant influenza viruses using reverse genetics techniques in avian, canine, equine or murine cells lines [155,156,157,158].

Influenza viruses require the presence of eight vRNA segments for efficient virus fitness and successful production of virion progeny. The initial description of influenza reverse genetics, originally established in 1999 for IAV [154,159], required the use of 12 plasmids to generate recombinant influenza viruses: four polymerase II (Pol II) protein expression plasmids, encoding the viral RdRp complex (PB2, PB1 and PA) and NP for vRNP reconstitution; and eight Pol I-driven plasmids for expression of the eight vRNA segments [154,159]. However, it was later described that only eight ambisense and/or bidirectional plasmids were needed for complete reconstitution of influenza viruses (Figure 3A) [150,151]. The eight plasmid-based rescue system is now the most common method for the generation of recombinant influenza viruses. Because fewer plasmids are required, the eight-plasmid approach is more successful than the initial twelve-plasmid reverse genetic technique. The core of the eight-plasmid rescue system is that each plasmid contains an “ambisense cassette” that includes RNA Pol I and/or II sequences, which drives the transcription of vRNAs (Pol I) and protein (Pol II) expression from the same viral cDNAs (Figure 3B). Using a similar technology in 2002, this reverse genetics technique allowed the recovery of IBV entirely from ambisense plasmids [13,160,161,162]. Now, IAV and IBV reverse genetics techniques are well established and commonly used in multiple research laboratories for different research purposes.

### 3.2. Reverse Genetics for the Investigation of Influenza Virus

Influenza reverse genetics techniques have had an important effect on expanding our knowledge of the molecular biology and pathogenesis of influenza viruses, allowing researchers to answer important questions in the biology of IAV and IBV that were not possible using conventional virological or biochemical procedures [12,144,147]. Scientists can now mutate specific nucleotides in the influenza viral genome to elucidate the nature of regulatory sequences or the contribution of specific amino acids to the function of influenza viral proteins. For instance, reverse genetics technologies have enabled the identification and characterization of the *cis*-acting elements required for virus cell entry, uncoating, genome replication and gene transcription, encapsidation, packaging and viral release [13,135,163,164,165]. Moreover, by engineering viral vectors suitable for the expression of foreign proteins in infected cells, multiple recombinant influenza viruses harboring reporter fluorescent and/or luminescent genes have been generated and used to identify antivirals or NAbs in vitro and/or in vivo [142,146,147]. Finally, reverse genetics have allowed the creation of single-cycle infectious IAVs (sciIAVs) that are restricted to one cycle of replication in parental cell lines. However, in complementing cell lines, sciIAVs can replicate efficiently and to levels comparable to WT forms of influenza viruses [144]. Highly virulent IAV have the potential to pose a greater human threat than many other Biosafety Level (BSL)-3 and BSL-4 pathogens because of their efficient transmission and limited therapeutic options [47]. Traditional immunological approaches (e.g., HAI or microneutralization assays) to identify the presence of IAV NAbs rely on the manipulation of live forms of viruses and need the use of special BSL conditions. Thus, novel approaches that allow the detection of viral NAbs without the use of live forms of IAVs and highly contained BSL laboratories would facilitate these serological assays. In this regard and because of their safety profile, sciIAVs represent an excellent alternative to identify new antivirals and/or NAbs [144]. Moreover, several sciIAVs have been shown to represent an excellent option for their implementation as safe, immunogenic and protective vaccines and/or vaccine vectors [69,144].

### 3.3. Generation of Recombinant Influenza Viruses Using Reverse Genetics Approaches

**Figure 4:**
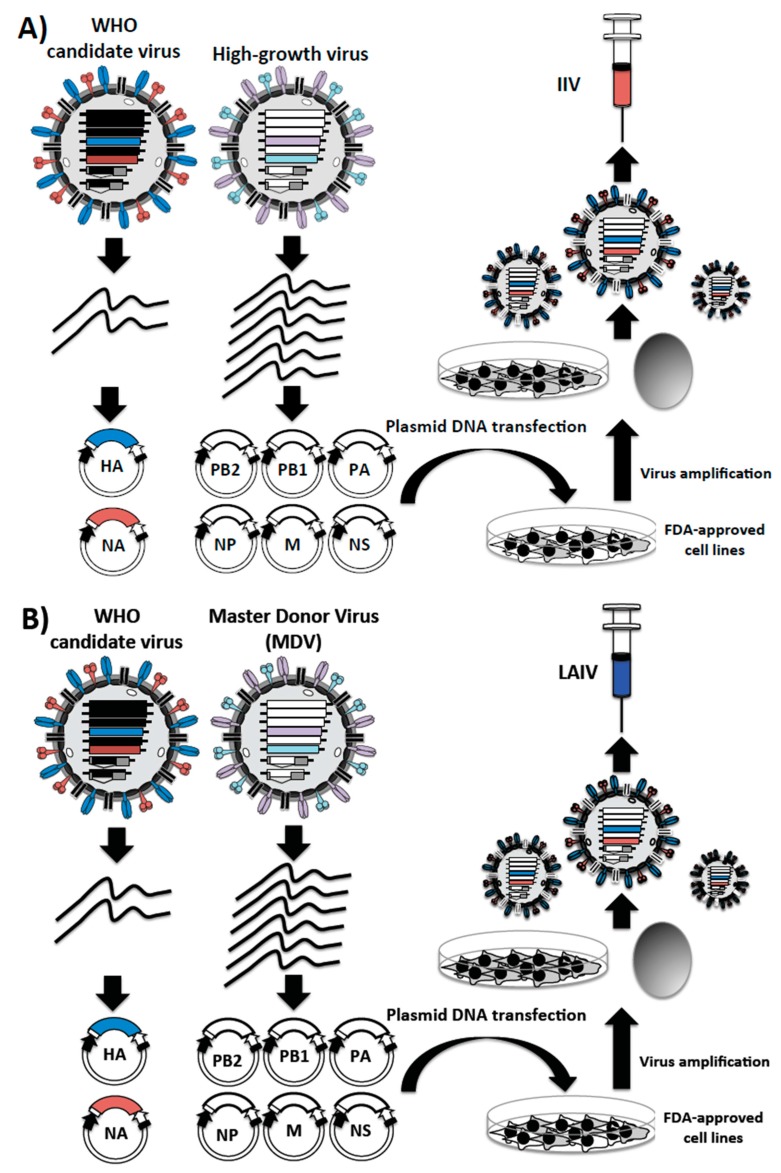
Reverse genetics approaches to generate influenza vaccines: For the development of reverse genetics, influenza vRNAs are cloned into the eight bi-directional plasmids. Transfection of the eight ambisense plasmids into permissible FDA-approved for vaccine production cell lines leads to the rescue of the recombinant influenza viruses containing the six internal genome segments from the high-growth virus (**A**) or from the MDV (**B**) and two genome segments (the HA and NA encoding segments) from WHO candidate strain for their use as IIV (**A**) or LAIV (**B**), respectively. Because the viruses are derived entirely from DNA, no selection system is needed to isolate the desired reassortant. The rescued viruses can be amplified and used as seed viruses for vaccine production.

Influenza plasmid-based reverse genetics represent a better alternative to circumvent the process of generating reassortant virus by co-infection of chicken embryonated eggs for the generation of influenza vaccines (Figure 2) [135]. Moreover, the generation of recombinant influenza viruses using plasmid-based reverse genetics approaches is simple, well established and currently in use in several laboratories around the world [139,166] (Figure 4). Briefly, the eight-ambisense plasmids are co-transfected into susceptible FDA-approved cells, and viable virus can be recovered from the tissue culture supernatants [139,166], then amplified in either embryonic eggs or FDA-approved for vaccine production cells (Figure 4).

### 3.4. Influenza Reverse Genetics for the Development of IIV

For many years, IIVs have been produced by reassortment in eggs (Figure 2) [90,98,122]. However, strains with the desired genotype (six internal genes of a high-growth virus and the HA and NA glycoproteins of the seasonal influenza virus) could be produced easily and more quickly by implementing reverse genetics approaches. This process would overcome the need of chicken embryonated eggs to generate the desired reassortant virus and, therefore, minimizing the time associated with the selection process of the reassortant IIV (Figure 4A). Moreover, the improvements in surveillance, as well as the de novo gene synthesis for the production of the HA and NA viral segments from the selected strains could reduce considerably the time of vaccine production. Although influenza reverse genetics could be useful for the production of vaccine seed strains, to date, it has not been possible to predict which gene segment(s) constellations are required for efficient growth of different vaccine viruses. Moreover, the time to produce vaccine viruses using reverse genetics versus the time needed to generate them using the classical reassortment approach could be a major consideration for the cost effectiveness in vaccine production and manufacturing. Thus, the traditional viral reassortment has remained preferred because it allows the generation of a number of diverse gene(s) combinations in order to select recombinant viruses with better fitness [98].

### 3.5. Influenza Reverse Genetics Approaches for the Development of Live-Attenuated Influenza Vaccines (LAIVs)

During the last few decades, considerable improvements have been accomplished in the development of influenza vaccines. However, novel approaches to increase the effectiveness of seasonal influenza vaccines are needed. Reverse genetics technologies have proven a valuable tool to develop reassortant strains for the production of LAIV candidates (Figure 4B). Currently, seed viruses containing six gene segments from the MDV A/Ann Arbor/6/60 (H2N2) and the HA and NA from the selected seasonal virus can be quickly generated using reverse genetics systems [98,131] (Figure 4B). The HA and NA from selected seasonal influenza viruses can be amplified by RT-PCR or quickly chemically synthetized and then cloned in ambisense plasmids used for virus rescue. This technology could speed up the development of new LAIVs, bypassing the need to isolate the exact virus reassortment in eggs (Figure 2).

### 3.6. Taking Advantage of Reverse Genetics for the Development of Novel Vaccines

The best way to combat influenza virus infection is to prevent it. Thus, an urgent need exists to develop novel and more effective influenza vaccines. Plasmid-based reverse genetic technologies have allowed the engineering of recombinant influenza viruses that contain single or multiple mutations in the viral genome, which can be potentially implemented as novel or improved vaccine approaches. In fact, several novel vaccine candidates have been developed with promising results in animal models of experimentation. Here, we review and discuss some of them.

#### 3.6.1. NS1 Truncated or Deficient Viruses as LAIVs

Because of NS1’s ability to hijack the host innate immune IFN-I response, a variety of potential vaccine strategies have been developed, which are based on the use of modified NS1 proteins as a means for virus attenuation [167,168,169,170,171,172]. Equine [170], swine [169,173,174], avian [167,175,176], canine [177] and human [178,179] IAVs with partial truncations in or deletions of the viral NS1 protein are all attenuated in vitro and in vivo [167,169,170,179]. Importantly, these recombinant IAVs can induce a protective immune response upon a single intranasal vaccination in mice [177,179,180,181], horses [170], pigs [169,173,174], birds [167,175,176] and macaques [178]; therefore, they represent excellent LAIV candidates to prevent IAV infections. In addition, a similar approach has been employed to develop attenuated IBVs with similar results [180]. Mice inoculated with the NS1-truncated or -deleted mutants elicited an antibody response and showed protection against WT virus challenge [180]. Thus, these NS1-truncated influenza A and B viruses represent excellent candidates as safe, immunogenic and protective LAIVs for multiple virus strains in different animal models.

#### 3.6.2. Codon-Deoptimized LAIVs

The genetic code in animals encodes for 20 different amino acids (aa) using 61 codons. This degeneracy of the genetic code allows amino acids, except tryptophan (W) and methionine (M), to be encoded by more than one synonymous codon [46]. Viruses, including influenza, rely on the host cell translation machinery to synthesize their viral proteins for the formation of infectious virus progeny. As an evolutionary consequence, viruses have modified their codon usage according to the host they infect [182]. Experimentally, protein synthesis can be downregulated by synthetically deoptimizing the codon usage of a gene [141,182]. The generation of recombinant viruses containing genes with deoptimized codons is now feasible [141,182], and their level of attenuation depends on the viral gene targeted and the number of codon changes made during the codon deoptimization process [182]. For influenza viruses, many regions in the viral genome cannot be altered because of their important role in viral replication and transcription (e.g., NCRs), packaging (e.g., packaging signals), the presence of multiple overlapping ORFs (e.g., segments 7 and 8), etc. [48]. To date, recombinant IAVs that have been attenuated using a codon-pair [183,184] or a codon bias [182] deoptimization approach to decrease expression levels of the viral PB1, HA and NP [183], NA and HA [184] or NS1 and NEP [182] have been generated. Importantly, influenza viruses generated by codon deoptimization showed similar viral replication kinetics to WT virus in MDCK cells, which is important for their effective use for vaccine production. However, to date, the ability of these recombinant IAVs containing codon-pair or codon bias deoptimized viral segments to replicate in eggs has not yet been evaluated. Importantly, all of the codon deoptimized IAVs were attenuated in mice and able to provide, upon a single immunization dose, protection against a lethal challenge with a WT form of the virus, showing that LAIVs were safe, immunogenic and protective. However, mice do not accurately reflect virus pathogenesis and immunological responses seen in humans [81] and do not have the same codon usage bias as humans. Therefore, studies aimed to demonstrate the safety, immunogenicity and protection efficacy of codon deoptimized recombinant IAVs in other well-established animal models of influenza (e.g., guinea pigs, ferrets or nonhuman primates) could lead to their implementation as LAIVs in future vaccinations [141].

#### 3.6.3. Single-Cycle Infectious Viruses as LAIVs

Other promising approaches for the development of LAIVs relate to the use of sciIAVs [144]. SciIAVs based on their safety profile, ability to elicit protective humoral and cellular responses and protective effectiveness represent a feasible alternative to current influenza vaccines for the treatment of influenza viral infections [69,144]. However, to date, no single-cycle infectious IBVs (sciIBV) have been reported. When delivered intranasally, sciIAV has been shown to be safe in a mouse model of influenza infection, without signs of illness or mortality [69,185,186,187,188,189]. Moreover, and similar to the current LAIVs, intranasal immunization with a single dose of sciIAVs elicited localized mucosal immune responses and recruitment of influenza-specific CD8 T-cells into the lungs of vaccinated animals [145,185,186,190], the latest being the main contributor of immunity against challenge with heterologous influenza viruses [69,190]. Importantly, sciIAVs protected mice against lethal influenza virus challenges [69,144]. Moreover, similar safety, immunogenicity and protection efficacy of sciIAVs were observed in ferrets [69]. Vaccination of pigs with sciIAV has also been shown to be immunogenic and protective against challenge with swine influenza viruses [191]. Moreover, sciIAVs expressing foreign genes represent an excellent option for their implementation as bivalent vaccines. For instance, sciIAVs expressing the surface protein A (PspA) of *Streptococcus pneumoniae*, the hemagglutinin-neuraminidase (HN) protein of HPIV-3 or the fusion (F) protein of respiratory syncytial virus (RSV) [192,193,194], were able to induced Abs against the foreign polypeptides and reduced the viral load of the heterologous pathogen while retaining their ability to protect against challenge with IAV. It is worth indicating that, for vaccine purposes, it is important to consider what influenza viral gene is replaced, since it was shown that sciIAV replication is required for protection [69,185,189]. Although substitution of the polymerase (PB2, PB1 and PA) segments may allow insertion of larger foreign genes, removing the viral polymerase from sciIAVs limits their ability to express more polymerase during the single-cycle round of infection, decreasing total viral antigens and, thus, limiting their protective efficacy. Thus, sciIAVs where the viral HA or NA has been removed were able to confer, upon a single immunization, protection against a lethal challenge with influenza. [190]. On the contrary, a single dose of a sciIAV where the viral PB2 has been removed was only as efficacious as an inactivated virus [189]. It is worth noting that sciIAVs combine the advantages (better immunogenic properties of the LAIV and the safety profile of the IIV) and circumvent the disadvantages (safety of the LAIV and poor immunogenicity of the IIV) of current influenza vaccine approaches [144]. While sciIAVs have been shown to be safe, immunogenic and protective against lethal challenges with wild-type forms of IAVs in animal studies, no human trials have yet been performed.

#### 3.6.4. LAIVs Based on the Rearrangement of the Influenza Viral Genome

More recently, the rearrangement of the influenza virus genome has been shown to have great potential for the development of improved LAIVs against influenza virus, as well as vaccine vectors against other pathogens [195,196].

Avian influenza virus subtypes H5N1 and H9N2 have pandemic potential [197,198]. However, H5N1 IIV induces limited adaptive immune responses, and in the case of LAIV, there are safety concerns about the possibility of reassortment between the viral segments in the LAIV and circulating H5N1 strains. To overcome these concerns, a bivalent LAIV against influenza A/Vietnam/120320/04 H5N1 and A/Guinea fowl/Hong Kong/WF10/1999 H9N2 was generated using viral genome rearrangement [195]. To that end, the NEP was removed from the NS viral segment of the H9N2 virus and substituted by the HA of the H5N1 virus. H9N2 NS1 and H5N1 HA were separated by the foot-and-mouth disease virus (FMDV) 2A autocleavage site to allow co-linear expression of both viral proteins. Then, NEP was cloned down-strain of the H9N2 PB1 segment separated by another FMDV 2A autocleavage site. The rearranged H9N2 virus expressing the H5N1 HA was able to provide complete protection against challenge with A/Vietnam/1203/2004 H5N1 and also against a potential pandemic H9:pH1N1 IAV reassortant virus in both mice and ferrets [195].

Segments 7 (M) and 8 (NS) of IAVs use an alternative splicing mechanism to express two different viral proteins from the same viral segment. Recently, we have generated recombinant influenza A/Puerto Rico/8/34 H1N1 viruses containing modified M and/or NS segments, in which the overlapping ORFs of the M1 and M2 viral proteins (M segment) and/or the NS1 and NEP proteins (NS segment) were separated with the porcine teschovirus 1 (PTV-1) 2A autocleavage site [196]. Recombinant viruses with a rearranged M segment were affected or impaired in replication in vitro at nonpermissive temperatures (37 and 39 °C, respectively), whereas high viral titers were obtained at permissive low temperatures (33 °C) [196]. Notably, viruses containing the M split segment were highly attenuated in vivo, but able to confer, upon a single immunization dose, complete protection against a lethal homologous challenge with wild-type PR8 [196]. Importantly, viruses with a reorganized M segment were able to confer better protection than a temperature-sensitive, LAIV PR8 virus [103,104,115,196]. These studies demonstrate that the rearrangement of the influenza viral genome can be used for the generation of safe, immunogenic and protective LAIVs.

#### 3.6.5. Other Approaches to Generate Influenza Vaccines

Current influenza vaccines induce immunity to the influenza virus strain-specific HA antigen and are not very effective against new pandemic viruses, given that HA is highly susceptible to frequent changes by antigenic drift and shift [12,44,45,46,74,75,76]. To overcome these drawbacks, different approaches aimed to develop a universal influenza vaccine able to induce cross-protective broadly neutralizing immunity against conserved viral antigens, such as the ectodomain of M2 (M2e) [199], the HA stalk domain [200] or NA [201], have been explored.

The M2e antigen is a linear peptide that is very well conserved across IAV strains. Although the mechanism of M2e-specific immunity is unclear, protective anti-M2 antibodies have been observed in multiple animal models, including mice, ferrets and primates [36]. However, these conserved antigenic targets need to be presented in a carrier system or conjugated to adjuvant molecules.

Promising results have been obtained with virus-like particles (VLPs), which are morphologically similar to the virus and present surface proteins in a highly immunogenic form. Because VLPs do not contain the viral genome, they are considered safer than viral vaccines, yet still induce strong humoral and cellular immune responses [202]. VLPs are most commonly made by expression of HA, NA and M1 [203,204], although HA and NA alone may be sufficient for VLP production [205]. Pushko et al. reported the generation of pandemic and seasonal VLPs combining three IAV subtypes. Both the pandemic-subtype VLP (incorporating H5N1, H7N2 and H2N3) and seasonal VLP (A/New Caledonia/20/1999 H1N1, A/New York/55/2004 H3N2 and B/Shanghai/361/2002) induced NAbs and protected ferrets from lethal virus challenges [206].

## 4. Conclusions and Future Direction

Reverse genetics approaches to generate recombinant viruses, including influenza, have been described for representative family members of negative-sense, single-stranded RNA viruses. These plasmid-based reverse genetics methods have provided scientists with a unique opportunity to study different aspects of the biology and pathogenesis of these viruses, both in vitro and in vivo, as well as to generate attenuated forms to be used as vaccines [135]. In this review, we discussed the use of reverse genetics for the generation of influenza vaccines with a special focus on LAIVs. In general, LAIVs are highly immunogenic, and immunization usually induces faster and substantially higher levels of both systemic and local mucosal antibody and T-cell responses, providing better protection than their inactivated counterparts. Prevention of influenza virus infection requires seasonal vaccinations, and identification of the correct virus subtype to include in the vaccine leaves little time for vaccine development, scale up and distribution. Although influenza vaccines work well most of the years, if a new viral variant emerges after the strain to be included in the vaccine has been selected, the efficacy of the vaccine would be sub-optimal for this new variant strain. This usually results in decreased efficacy of the vaccine. Reverse genetics approaches have also allowed the development of live-attenuated viruses that could be implemented, in the near future, as LAIVs. These new advances in reverse genetics approaches are reducing the potential time of recovery and production from months to weeks and represent an excellent alternative for the rapid development and implementation of LAIVs for the treatment of both seasonal and potentially pandemic influenza strains.

## Abbreviations

IAV	Influenza A virus
IBV	Influenza B virus
vRNA	Viral RNAs
vRNP	Viral ribonucleoprotein
NAb	Neutralizing antibody
ts	Temperature-sensitive
ca	Cold-adapted
att	Attenuated
PB2	Polymerase basic 2
PB1	Polymerase basic 1
PA	Polymerase acid
HA	Hemagglutinin
NA	Neuraminidase
NP	Nucleoprotein
NS1	Non-structural protein 1
ORF	Open reading frame
NCR	Non-coding region
IFN-I	Type I interferon
LAIV	Live attenuated influenza vaccine
IIV	Influenza inactivated vaccine
VLP	Virus-like particle
WHO	World Health Organization
